# Supplementary material for: Ginsenosides and Polysaccharides from Ginseng Co-Fermented with Multi-Enzyme-Coupling Probiotics Improve In Vivo Immunomodulatory Effects
Source: Nutrients. 2023 May 23;15(11):2434. doi: 10.3390/nu15112434 (PMC10254733; doi:10.3390/nu15112434)
Supplement: Supplementary file 1 [file nutrients-15-02434-s001.zip › nutrients-2343570-supplementary.pdf]

Table S1 strains and sources

Strain	Source
Streptococcus thermophilus Str-002	Directed Vat Set
Lactobacillus plantarum CCFM 9059	CCFM
Lactobacillus rhamnosus ATCC 10863	ATCC
Bacillus amyloliquefaciens JD-001	Preserved in our lab

Table S2 Animal experiment group

Group (abbr.)	Group
NC	Normal Control
MC	Model Control
GC	Ginseng Control
LE	*Lactobacillus rhamnosus*-Enzyme
LF	*Lactobacillus rhamnosus* Fermentation
BE	*Bacillus amyloliticus*-Enzyme
BF	*Bacillus amyloliticus* Fermentation
LF&BF	*Lactobacillus rhamnosus* and *Bacillus amyloliticus* Fermentation
PC	Positive Control

Table S3 Factors and level of orthogonal test

Level	Factors (additive contents %)		
	Cellulase (A)	Compound pectinase X (B)	Flavor protease (C)
1	0.15	0.40	0.10
2	0.20	0.60	0.15
3	0.25	0.80	0.20

Table S4 Analysis of variance in orthogonal test of *Lactobacillus rhamnosus*

Independent variables	Type III SS	DF	MS	F value	SD
Cellulase	1.0×10^{16}	2	5.2×10^{15}	284.629	0.004
Compound pectinase X	10.0×10^{14}	2	5.0×10^{14}	27.124	0.036
Flavor protease	4.4×10^{14}	2	2.2×10^{14}	11.897	0.078
Error	3.7×10^{13}	2	1.8×10^{13}		

SS: Sum of Squares, DF: Degree of Freedom, MS: Mean Square, SD: Significant Difference

Table S5 Analysis of variance in orthogonal test of *Bacillus amyloliquefaciens*

Independent variables	Type III SS	DF	MS	F value	SD
Cellulase	3.0×10^{14}	2	1.5×10^{14}	2.907	0.256
Compound pectinase X	2.5×10^{16}	2	1.2×10^{16}	244.087	0.004
Flavor protease	3.6×10^{14}	2	1.8×10^{14}	3.561	0.219
Error	1.0×10^{14}	2	5.1×10^{13}		

SS: Sum of Squares, DF: Degree of Freedom, MS: Mean Square, SD: Significant Difference

Table S6 The viable count in ginseng freeze-dried powder

Group	Viable count (CFU/g)*			
Multi-enzyme coupling *Lactobacillus rhamnosus*	3.9×10^{10}	3.4×10^{10}	3.6×10^{10}	3.0×10^{10}
Multi-enzyme coupling *Bacillus amyloliquefaciens*	2.2×10^{11}	1.9×10^{11}	1.9×10^{11}	1.8×10^{11}

* Four parallel experiments
